# Unique rumen micromorphology and microbiota–metabolite interactions: features and strategies for Tibetan sheep adaptation to the plateau

**DOI:** 10.3389/fmicb.2024.1471732

**Published:** 2024-10-09

**Authors:** Qianling Chen, Yuzhu Sha, Xiu Liu, Yanyu He, Xiaowei Chen, Wenxin Yang, Min Gao, Wei Huang, Jiqing Wang, Jianwen He, Lei Wang

**Affiliations:** ^1^College of Animal Science and Technology, Gansu Agricultural University, Lanzhou, China; ^2^School of Fundamental Sciences, Massey University, Palmerston North, New Zealand; ^3^College of Animal Husbandry and Veterinary Science and Technology, Gansu Vocational College of Agricultural, Lanzhou, China; ^4^Zhangye City Livestock Breeding and Improvement Workstation, Zhangye, China

**Keywords:** Tibetan sheep, Hu sheep, 16S rRNA, metabolomics, VFAs, transporter genes

## Abstract

The rumen microbiota—a symbiont to its host and consists of critical functional substances—plays a vital role in the animal body and represents a new perspective in the study of adaptive evolution in animals. This study used Slide Viewer slicing analysis system, gas chromatography, RT-qPCR and other technologies, as well as 16S and metabolomics determination methods, to measure and analyze the microstructure of rumen epithelium, rumen fermentation parameters, rumen transport genes, rumen microbiota and metabolites in Tibetan sheep and Hu sheep. The results indicate that the rumen nipple height and cuticle thickness of Tibetan sheep are significantly greater than those of Hu sheep (*p* < 0.01) and that the digestion and absorption of forage are greater. The levels of carbohydrate metabolism, lipid metabolism, and protein turnover were increased in Tibetan sheep, which enabled them to ferment efficiently, utilize forage, and absorb metabolic volatile fatty acids (VFAs). Tibetan sheep rumen metabolites are related to immune function and energy metabolism, which regulate rumen growth and development and gastrointestinal homeostasis. Thus, compared with Hu sheep, Tibetan sheep have more rumen papilla and cuticle corneum, and the synergistic effect of the microbiota and its metabolites is a characteristic and strategy for adapting to high-altitude environments.

## 1 Introduction

Tibetan sheep constitute one of the three major rough-wooled sheep breeds in China (the others include Kazakh sheep and Mongolian sheep), are distributed mainly in the alpine areas of Tibet and Qinghai on the Tibetan Plateau at altitudes of more than 2,000 m, are important livestock germplasm resources on the Tibetan Plateau, and have low oxygen, rough feeding, and disease resistance, In high-altitude environments, Tibetan sheep have developed particular biological habits and digestive, and metabolic mechanisms adapted to the alpine climate, demonstrating strong environmental adaptability. The rumen, a unique digestive organ in ruminants, is a complex ecosystem that plays a vital role in the metabolism, immune system, and health of the host (Lin et al., 2019). The rumen is inhabited by a wide variety of microorganisms, including bacteria, fungi, and protozoa These microorganisms make up a large microbiota that converts the ingested feed and other nutrients into microbial proteins and volatile fatty acids (VFAs) and synthesizes amino acids and proteins that cannot be synthesized by the animal itself, thus increasing the metabolic reservoirs of the host (Hess et al., 2011; Muegge et al., 2011). Therefore, rumen microorganisms play a vital role in the performance and health of ruminants. VFAs, as significant metabolites of rumen microorganisms, can provide approximately 75% of the energy requirements of ruminants (Bergman, 1990). They are also signaling molecules that regulate rumen epithelial cell proliferation and immune responses (Hosseinkhani et al., 2021; Li et al., 2021). Therefore, understanding the factors that affect the rumen microbiota and its metabolite composition will help reveal the biological mechanisms by which microorganisms and metabolites alter the body’s adaptability and elucidate the mechanisms of host–microbiota–metabolite interactions, thereby providing new coping strategies and methods.

Interactions between the gut microbiota and between the gut microbiota and the host are an essential part of the dynamic equilibrium of the gut microbiota, and changes in metabolites are closely related to changes in the gut microbiota. Factors that influence microbial composition and function include temperature, host species, diet, exercise, age, and geographic location (Wang et al., 2019). Still, these effects account for only 10 ~ 20% of microbial diversity, and the vast majority of microbial diversity between individuals remains unexplained. Therefore, the study of interactions between the integrated host genome, transcriptome, metabolome, and microbiome has received increasing attention. The gut microbiota and host coevolution coregulate host phenotypes. Under the same environmental feeding conditions, the composition of the gut microbiota of different species of Hydra varies widely. The composition of the gut microbiome of each of the two species of Hydra in the wild is very similar to that of the same species under laboratory conditions in that the host selectively determines the gut microbiome (Fraune and Bosch, 2007). Host genetic and environmental factors combine to influence the gut microbiota; In a study of two family lines of chickens with high and low body weights, the host genetic background has an essential influence on the composition and abundance of gut microorganisms. Some gut microbiota showed significant heritability and genetic correlation (Meng et al., 2014). In addition, there is evidence that host factors (such as breed, genetic variation, etc.) influence the composition and function of the bovine rumen microbiome (Zang et al., 2022). For example, the abundance of the rumen microbiota associated with CH_4_ emissions in dairy cows is influenced by host genotype to some extent (Difford et al., 2018; Zhang et al., 2020). Host genetic factors also influence rumen microbiota formation in beef cattle (Abbas et al., 2020). In recent years, elucidating the mechanisms related to host biological indicators and phenotypic traits via multiomics has become a research hotspot (Wang et al., 2019). Zhang et al. reported that yaks and Tibetan sheep can adapt to extreme environments through coevolution of the rumen microbiota and host genes (Zhang et al., 2016). The regulatory mechanisms by which the rumen epithelial genome of Tibetan sheep interacts with the microbiota and its metabolites to adapt to the plateau cold season have been investigated previously via RNA sequencing (RNA-Seq) and microbiome analyses (Liu et al., 2022). All these studies indicate that microorganisms metabolically regulate the host phenotype. In addition, Tibetan sheep live at high altitudes throughout the year and have a strong adaptive ability to extreme plateau environments. In contrast, Hu sheep live at low altitudes throughout the year and have the advantages of high fertility, early sexual maturity, roughage tolerance, environmental adaptability (EEr et al., 2020; Li et al., 2022), and good adaptability to semiarid and semidesert areas, and they have been widely introduced in Northern China and high-altitude regions (Guo et al., 2023). However, only a few studies have been performed on rumen microbial–host interactions regulating plateau adaptation in Tibetan sheep and Hu sheep. Therefore, in this study, we compared the morphology of the rumen epithelium and fermentation function with microorganisms and their metabolites by using Tibetan sheep and Hu sheep reared in the same plateau environment to analyze and explore the phenotypic and genetic differences in plateau adaptation between Tibetan sheep and Hu sheep, to reveal the mechanisms by which the Tibetan sheep rumen microbiota and metabolites on rumen fermentation function and host interactions, and providing a basis for the study of plateau adaptation in Tibetan sheep.

## 2 Materials and methods

### 2.1 Experimental animals and sample collection

The experimental samples were selected from the same herder’s flock in Hezuo City, Gannan Tibetan Autonomous Prefecture, Gansu Province (103°E, 35°N), which has an altitude of 3,000 m, a humid and high-altitude climate, and a relatively fragile ecological environment. Six Tibetan sheep and 6 Hu sheep weighing 34 kg (± 0.5 kg) and aged 1 year (± 1 month) were selected for the experiment. The experimental sheep exhibited regular feeding and rumination, shiny fur, good physical condition, oval-shaped feces, and adherence to each other after landing, indicating good health. They were all in the local area’s traditional natural grazing management state. The types of forage mainly included *Poa poophagorum* Bor, *Poaceae*, *Carex coninux*, *Argentina anserina*, and *Geranium platyanthum* Duthie, without any supplementary feeding. The nutritional components of forage are shown in Supplementary Table S1. The test sheep were slaughtered at Xinling Livestock Product Development Co., Ltd. in Hezuo City, Gannan Tibetan Autonomous Prefecture, Gansu Province, and the rumen contents and tissues were collected. Before grazing in the morning, the rumen fluid was collected via a sheep gastric tube-type rumen sampler. Three tubes were collected from each sheep, and the collected rumen fluid was quickly put into a liquid nitrogen tank for freezing, then returned to the laboratory for preservation at −80°C and subsequent 16S rRNA analysis and metabolite identification. Immediately after the jugular vein was bled to death, the rumen was isolated, and a block of rumen vesicle tissue (1 cm^2^) was collected, lightly rinsed with saline to remove the contents, and fixed in 4% paraformaldehyde for histomorphological analysis. At the same time, a small piece of the rumen vesicle was clipped, and the contents were rinsed off rapidly with precooled saline at 4°C and PBS buffer, after which the epithelial tissues were separated with blunt scissors and rapidly placed in liquid nitrogen for subsequent total RNA extraction.

### 2.2 Morphological analysis

Rumen ventral sac tissues were fixed in 4% paraformaldehyde for 24 h. The fixed tissue samples were immersed in dehydrating agents in the following order: (1) 50% alcohol for 2 h; (2) 75% alcohol for 2 h, or could overnight; (3) 85% alcohol for 2 h; and (4) 95% alcohol for 1 h. The dehydrated tissue samples were washed with alcohol and then quickly transferred to xylene for clearing, and the tissue was transparent after approximately 15 min. After 15 min, the tissue was transparent. The transparent samples were transferred to melted paraffin, allowing the paraffin to contact and immerse the tissue fully, and then embedded in a wax block after 2 h. The wax block was fixed in a sectioning folder. After the wax block in the sectioning folder was fixed, the tissues were sectioned at a thickness of 3 μm, and the sections were spread and mounted in distilled water at 45°C and dried by baking at 60°C. The paraffin sections were stained in the following order: (1) Xylene I dewaxing, 10 ~ 15 min; (2) xylene II dewaxing, 1 ~ 2 min; (3) xylene: Anhydrous ethanol = 1:1 mixture, 1 ~ 2 min; (4) anhydrous ethanol, 1 ~ 2 min; (5) downward-gradient alcohol hydration: 95, 85, 75, and 50% alcohol, 1 ~ 2 min each; (6) gentle washing with distilled water, 1 ~ 2 min; (7) hematoxylin staining, 10 ~ 15 min; (8) gentle washing with distilled water to remove excess dye; (9) alcohol separation in hydrochloric acid, 30 ~ 60 s; (10) rinsing with tap water for bluing, 15 ~ 20 min; (11) gentle washing with distilled water, 1 ~ 2 min; (12) dehydration in upward-graded alcohols: 1 ~ 2 min each in 50%, 75, and 85% alcohols; (13) eosin staining, 2 ~ 3 min; (14) upward gradient alcohol dehydration: 75, 85, 95% alcohol, and anhydrous ethanol dehydration, 1 ~ 2 min each; and (15) xylene I, xylene II transparent, 15 min. Finally, neutral gum sealing, drying, observation under the microscope, and imaging were performed. Rumen muscle layer thickness, nipple height, nipple width, cuticle thickness, granular layer thickness, spinous layer thickness, and basal layer thickness were determined via Slide Viewer.

### 2.3 16S ribosomal RNA (rRNA) sequencing analysis of rumen microorganisms

Microbiome DNA was extracted from the rumen fluid of Tibetan sheep and Hu sheep using the MN NucleoSpin 96 Soi bacterial DNA extraction kit (Omega, Shanghai, China). Polymerase chain reaction (PCR) amplification of the V3–V4 region of the highly variable region of the 16S rRNA gene was performed using universal primers (forward primer 338F: 5’-ACTCCTACGGGGAGGCAGCA-3’ and reverse primer 806R: 5’-GGACTACHVGGGTWTCTAAT-3’). For library sequencing, small fragment libraries were constructed using a two-step library construction method with double-end sequencing, the amplified products were analyzed by library sequencing on the Illumina MiSeq 2,500 platform (Illumina, San Diego, CA, USA), and bioinformatics analysis was performed using BMKCloud^1^ (accessed on 01 June 2024). To evaluate the quality of the raw data obtained from sequencing, paired-end splicing (FLASH version 1.2.7), filtering (Trimmomatic version 0.33), and chimeras (UCHIME version 4.2) were removed to obtain optimized sequences (Tags). The operational taxonomic units (OTUs) were obtained using USEARCH software. OTU classification and annotation were analyzed using the Silva (Bacteria) taxonomy database for further taxonomic analysis, and the community structure at different taxonomic levels (phylum, class, order, family, genus, and species) was obtained. The species diversity was analyzed via *α*-diversity, the α-diversity indices Ace, Chao1, Shannon, and Simpson were obtained, and a sample rarefaction curve was drawn. Furthermore, *β*-diversity analysis was used to obtain principal coordinates components (principal coordinate analysis [PCoA]) and boxplots based on multiple distances according to the distance matrix (Anosim). Line discriminant analysis effect size (LEfSe) analysis was used to find biomarkers with significant differences between groups. Metastats was used to conduct *t*-tests on species abundance data between groups. The species that caused the difference in the composition of the two groups of samples according to the *q* value were screened out, and 16S gene function analysis was performed using the Kyoto Encyclopedia of Genes and Genomes (KEGG) and Clusters of Orthologous Groups of proteins (COG) (Sha et al., 2022).

### 2.4 Metabolomic analysis of rumen microorganisms

Microbial metabolic profiling of rumen fluid from Tibetan sheep and Hu sheep (*n* = 12) was performed using a liquid chromatography–mass spectrometry (LC–MS) platform. After the samples were thawed at room temperature, 100 μL of each sample was weighed, and 500 μL of the extraction solution (methanol/acetonitrile volume ratio of 1:1, internal standard concentration of 2 mg/L) containing the internal standard (1,000:2) was added and vortexed for 30 s. The samples were then sonicated in an ice–water bath for 10 min, stood at −20°C for 1 h, and then centrifuged at 4°C at 12,000 rpm for 15 min. Then, 500 μL of the supernatant was placed in an EP tube, and the extract was dried in a vacuum concentrator, 150 μL of extract (acetonitrile−water volume ratio of 1:1) was added to the dried metabolite for redissolution, vortexing was continued for 30 s, sonication was performed for 10 min in an ice–water bath, and centrifugation was performed for 15 min at 12,000 rpm at 4°C. Finally, 120 μL of the supernatant was removed from a 2-ml injection bottle, and 10 μL of each sample was mixed with a QC sample for testing. The LC–MS system for metabolomics analysis was composed of a Waters Acquity I-Class PLUS Ultra High-Performance Liquid Tandem Waters Xevo G2-XS QToF High-Resolution Mass Spectrometer, and the column used here was purchased from a Waters Acquity UPLC HSS T3 column (1.8 μm 2.1 × 100 mm). The samples were eluted using positive (ESI+) and negative (ESI−) mobile phases consisting of water and 5% acetonitrile, 0.1% formic acid as solvent A and acetonitrile and 0.1% formic acid as solvent B at flow rates of 0.35 mL/min and 400 μL/min, respectively. The subsequent mobile phase (A:B) elution gradient was 0–0.25 min 98–2%, 10.0–13.0 min 2–98%, and 13.1–15.0 min 98–2%, followed by an ion source temperature of 150°C and a desolvation temperature of 500°C. The flow rates of the reverse gases and desolvent gas were 50 and 800 L/h, respectively. The raw data collected via MassLynx (version 4.2) were processed via Progenesis QI software. The Progenesis QI software, online databases such as METLIN and the self-built databases of BMG were used for metabolite identification according to sample type. BMKCloud was used to conduct a subsequent bioinformatics analysis of the identified metabolites. The screening criteria for differentially abundant metabolites were FC > 1, *p* < 0.05, and VIP > 1, and the differentially abundant metabolites were analyzed by KEGG functional annotation and enrichment.

### 2.5 Measurement of rumen VFAs

Thaw rumen fluid samples at room temperature, centrifuge at 15,000 rpm for 15 min at 4°C, aspirate 1 mL supernatant into a 1.5 mL centrifuge tube, add 0.2 mL of 25% metaphosphoric acid solution containing internal standard 2 EB, mix well and leave on ice for more than 30 min. Centrifuge at 15,000 rpm for 15 min, aspirate the supernatant with a 2 mL syringe and filter the supernatant with a 0.22-μm filter tip. The supernatant was aspirated with a 2-ml syringe and filtered with a 0.22-μm filter tip, and the resulting filtrate was transferred to a new collection tube for testing.

An Agilent 7890B gas chromatograph was used for the determination. The chromatographic column was an AT-FFAP capillary column (30 m × 0.32 mm × 0.50 μm), the temperature of the inlet (SSL): 250°C; the temperature of the detector (flame ionization detector [FID]): 250°C; the carrier gas was high-purity nitrogen (99.999%), the total pressure was 100 kPa, and the splitting ratio was 5:1; the gas flow rate was as follows: air: 400 mL·min^−1^, H_2_: 35 mL·min^−1^, and N_2_: 40 mL·min^−1^; injection volume was 1 μL; and the heating procedure was as follows: 120°C held for 3 min, 10°C·min^−1^ heating to 180°C, held for 1 min.

### 2.6 Measurement of rumen VFA transporter-related gene expression

Total RNA was extracted from the rumen tissues of Tibetan sheep and Hu sheep using the TRIzol reagent method (DP762-T1C; Life Technologies, CA, USA). The RNA concentration and purity were determined using an ultramicro spectrophotometer (Therm Nano Drop-2000; Thermo Scientific, MA, USA), and the OD260:OD280 ranged 1.8–2.1, indicating that the purity of the extracted RNA was good. The integrity of the extracted RNA was examined using an agarose gel electrophoresis instrument (Agient2100, LabChip GX), and three single and well-defined bands were visualized using an agarose gel imaging system (Ready Agarose, Bio-Rad, Hercules, CA, USA), indicating that the integrity of the extracted RNA was good. cDNA synthesis was performed using a reverse transcription kit (HiScript® II Q RT SuperMix for qPCR; Nanjing, China). The primers for *anion exchanger 2* (*AE2*), *downregulated in adenoma* (*DRA*), *monocarboxylic acid transporter protein1* (*MCT1*), *monocarboxylic acid transporter protein* (*MCT4*), *Na^+^/H^+^ exchanger1* (*NHE1*) and *Na^+^/H^+^ exchanger2* (*NHE2*) genes were designed using Primer5.0 software (*β-actin* was used as an internal reference gene; see Supplementary Table S2 for detailed primer information). The relative expression of genes involved in the transport of VFAs in the rumen tissues of Tibetan sheep and Hu sheep was investigated using an Applied Biosystems Q6 real-time fluorescence quantitative PCR instrument. Real-time quantitative PCR (RT-qPCR) was performed using a 20-μl reaction system with the following reaction conditions: predenaturation at 95°C for 30 s; cycling at 95°C for 10 s and 60°C for 30 s for 40 cycles; and lysis curve (95°C for 15 s, 60°C for 60 s, and 95°C for 15 s). The relative gene expression was calculated by correcting for *β-actin* as an internal reference gene, and the data were analyzed by the 2^−∆∆CT^ method.

### 2.7 Statistical analysis of the data

The experimental data were initially organized using Excel 2016, and the analyzed data were expressed as “mean ± standard error” with a statistical significance level of *p* < 0.05. Then the data were analyzed using the Statistical Package for the Social Sciences (SPSS) version 24.0 software. The content of VFAs was analyzed using an independent samples *t*-test, and the relative expression of transporter genes was analyzed using the 2^−∆∆CT^ method. Correlation analyses were performed using Spearman’s correlation test with a screening criterion of *p* < 0.05.

## 3 Results

### 3.1 Morphological structure of the rumen epithelium in Tibetan sheep and Hu sheep

**Figure 1:**
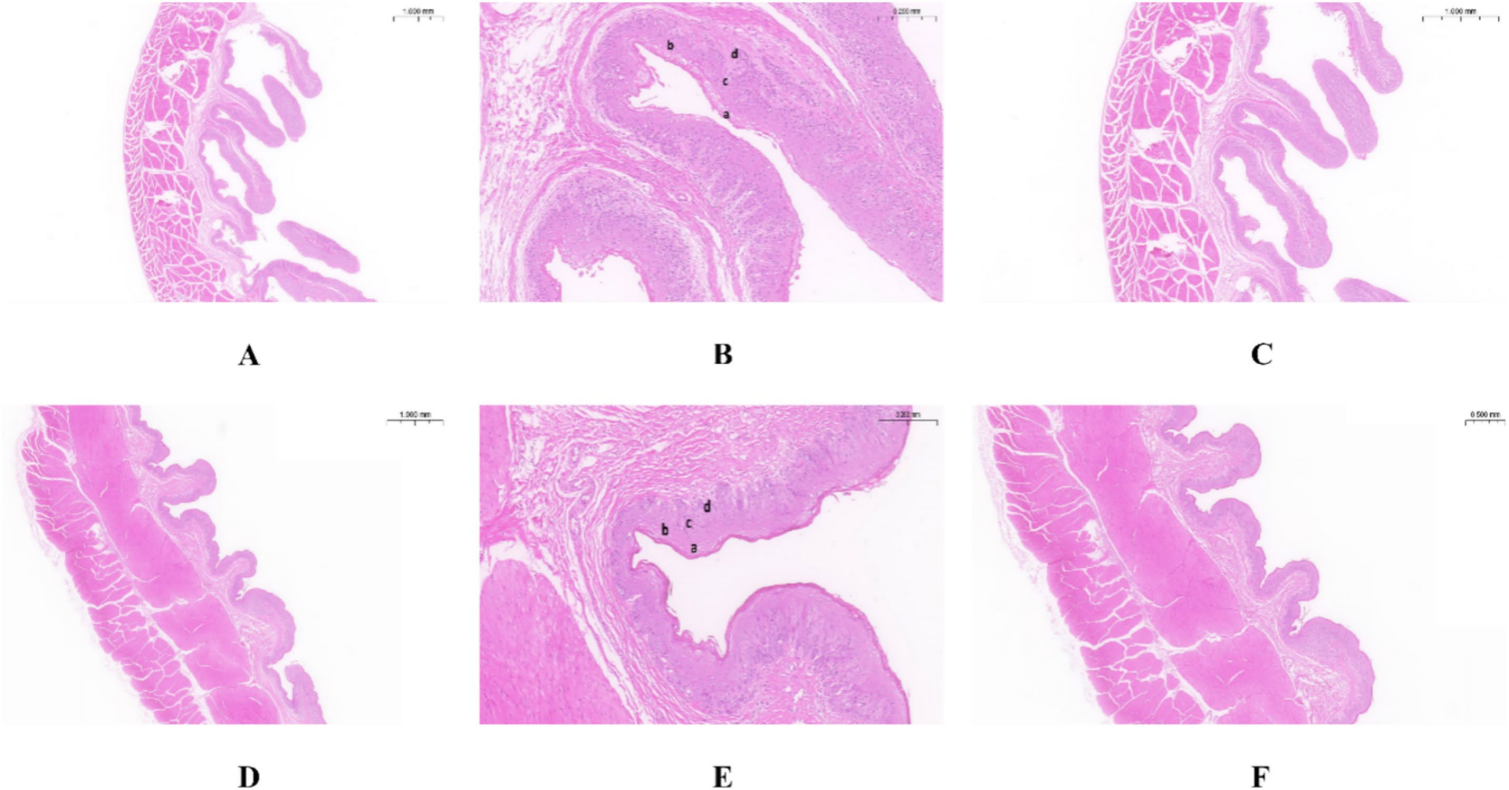
Morphology of rumen epithelial tissue of Tibetan sheep and Hu sheep. **(A–C)** The morphology of rumen epithelial tissue of Tibetan sheep at 1×, 10×, and 2× magnifications, respectively; **(D–F)** The morphology of rumen epithelial tissue of Hu sheep at 1×, 10×, and 2× magnifications, respectively. a: Cuticle; b: Granular layer; c: Spinous layer; d: Basal layer.

As shown in Table 1, the nipple height of the rumen epithelium of Tibetan sheep is significantly greater than that of Hu sheep (*p* < 0.01), and the nipple width of Hu sheep is significantly greater than that of Tibetan sheep (*p* < 0.05). The sheep rumen epithelial layer is divided from outside to inside into the cuticle (a), granular layer (b), sphenoid layer (c), and basal layer (d) (Figures 1B,E). As shown in Figure 1, the differences in the rumen epithelial layer between Tibetan sheep and Hu sheep included a significantly greater thickness of the granular layer and sphenoid layer in Hu sheep than in Tibetan sheep (*p* < 0.01), a significantly greater thickness of the cuticle layer in Tibetan sheep than in Hu sheep (*p* < 0.01), and a non-significant difference in the thickness of the basal layer (*p* > 0.05). In addition, the differences in the morphology of the muscular layer of the rumen wall between Tibetan sheep and Hu sheep, in which the thickness of the muscular layer is significantly greater than that of Tibetan sheep (*p* < 0.01), and the number of connective tissues in the muscular layer of the Hu sheep is greater than that of the Tibetan sheep (Figures 1A,C,D,F).

**Table 1:** Morphological analysis of rumen epithelium of Tibetan sheep and Hu sheep.

Project/um	Tibetan sheep	Hu sheep	*p*-value
Muscle thickness	1459.72 ± 79.58	1964.96 ± 155.48	<0.001
Nipple height	1926.02 ± 285.71	860.76 ± 290.28	<0.001
Nipple width	422.48 ± 16.86	541.70 ± 74.46	0.008
Cuticle thickness	32.46 ± 6.94	12.76 ± 1.99	<0.001
Granular layer thickness	18.20 ± 3.88	27.64 ± 1.41	0.001
Sphenoid layer thickness	27.18 ± 4.40	47.56 ± 5.58	<0.001
Basal layer thickness	29.58 ± 3.44	31.18 ± 2.27	0.411

### 3.2 Sheep rumen 16S measurement

**Figure 2:**
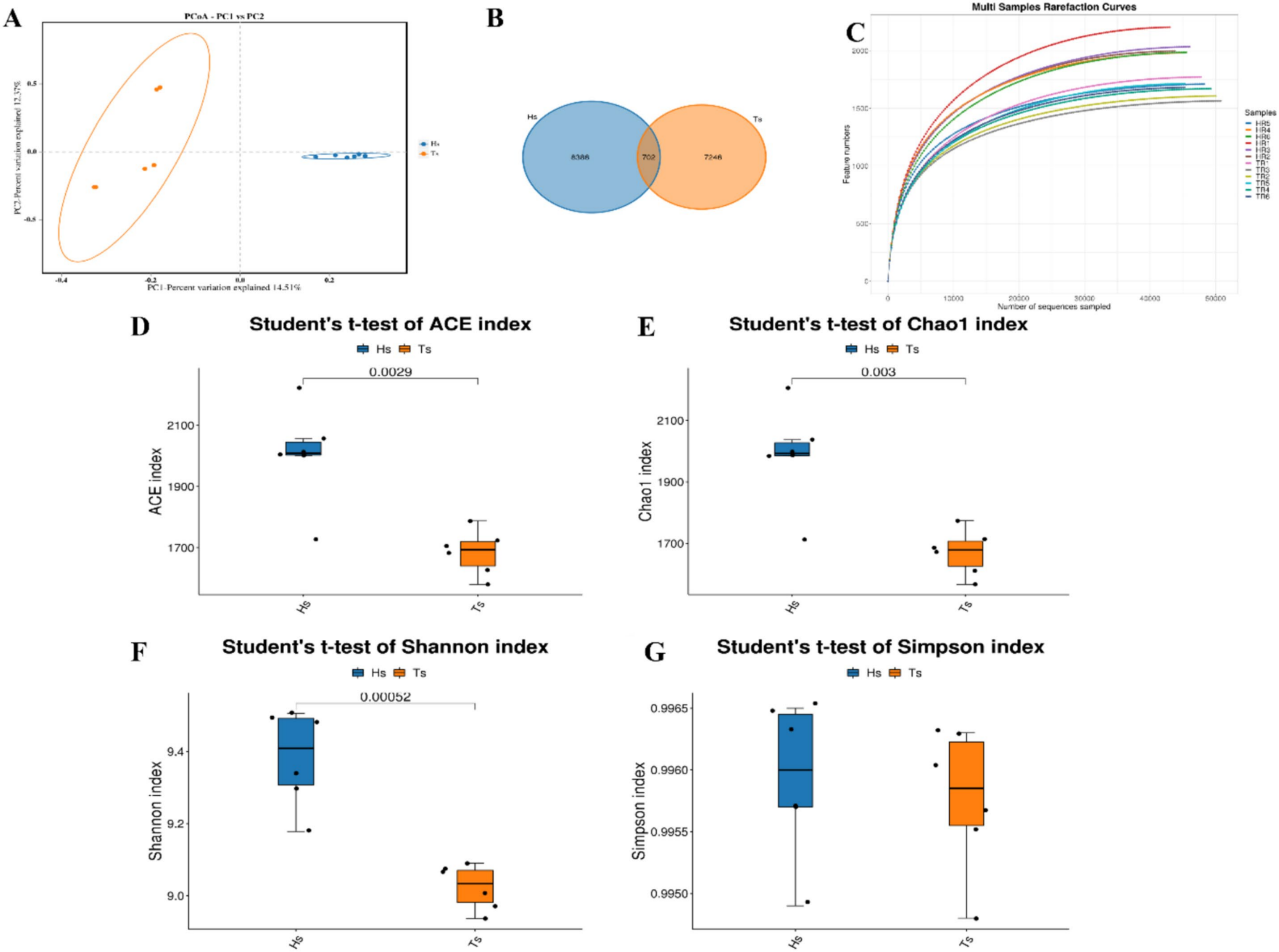
Analysis of microbial diversity in Tibetan sheep and Hu sheep. **(A)** Scatter plot of principal coordinates components (PCoA)-score showing similarity of the microbiological composition of Tibetan sheep and Hu sheep based on UniFrac distance, principal coordinates (PCs) 1 and 2 explained 14.51 and 12.37% of the variance, respectively. **(B)** OTU Venn diagram. **(C)** Sample dilution curve. **(D–G)** Microbial diversity indicators—**(D)**: ACE index; **(E)**: Chao1 index; **(F)**: Shannon index; and **(G)**: Simpson index.

16S rRNA sequencing of the rumen fluid from Tibetan sheep and Hu sheep (*n* = 12) and PCoA reveal that there are significant differences in the rumen microbes between Tibetan sheep and Hu sheep, with good within-group reproducibility (Figure 2A). A total of 16,334 OTUs are obtained, including 7,246 OTUs in Tibetan sheep and 8,386 OTUs in Hu sheep (Figure 2B). The number of OTUs specific to Hu sheep is significantly greater than that specific to Tibetan sheep (*p* < 0.05). Dilution curves are used to verify that the amount of sequencing data is sufficient to reflect the diversity of species in the samples and the abundance of species in the samples. As shown in Figure 2C, the curve flattens at 40,000 Reads, indicating that the amount of sequencing data is sufficient. To compare their differences more systematically, the rumen microbial compositions are analyzed. *α*-Diversity analysis reveals that the ACE, Chao1, and Shannon indices in the rumen microorganisms of Hu sheep are significantly greater than those in the rumen microorganisms of Tibetan sheep (*p* < 0.05), and the difference in the Simpson index is not significant (*p* > 0.05) (Figures 2D–G). The microbial diversity index of Hu sheep is significantly higher than that of Tibetan sheep. The higher the α-diversity is, the more complex and stable the composition of the gut microbiota, indicating that Hu sheep are more resistant and adaptable to external interference, which is beneficial for host health.

**Figure 3:**
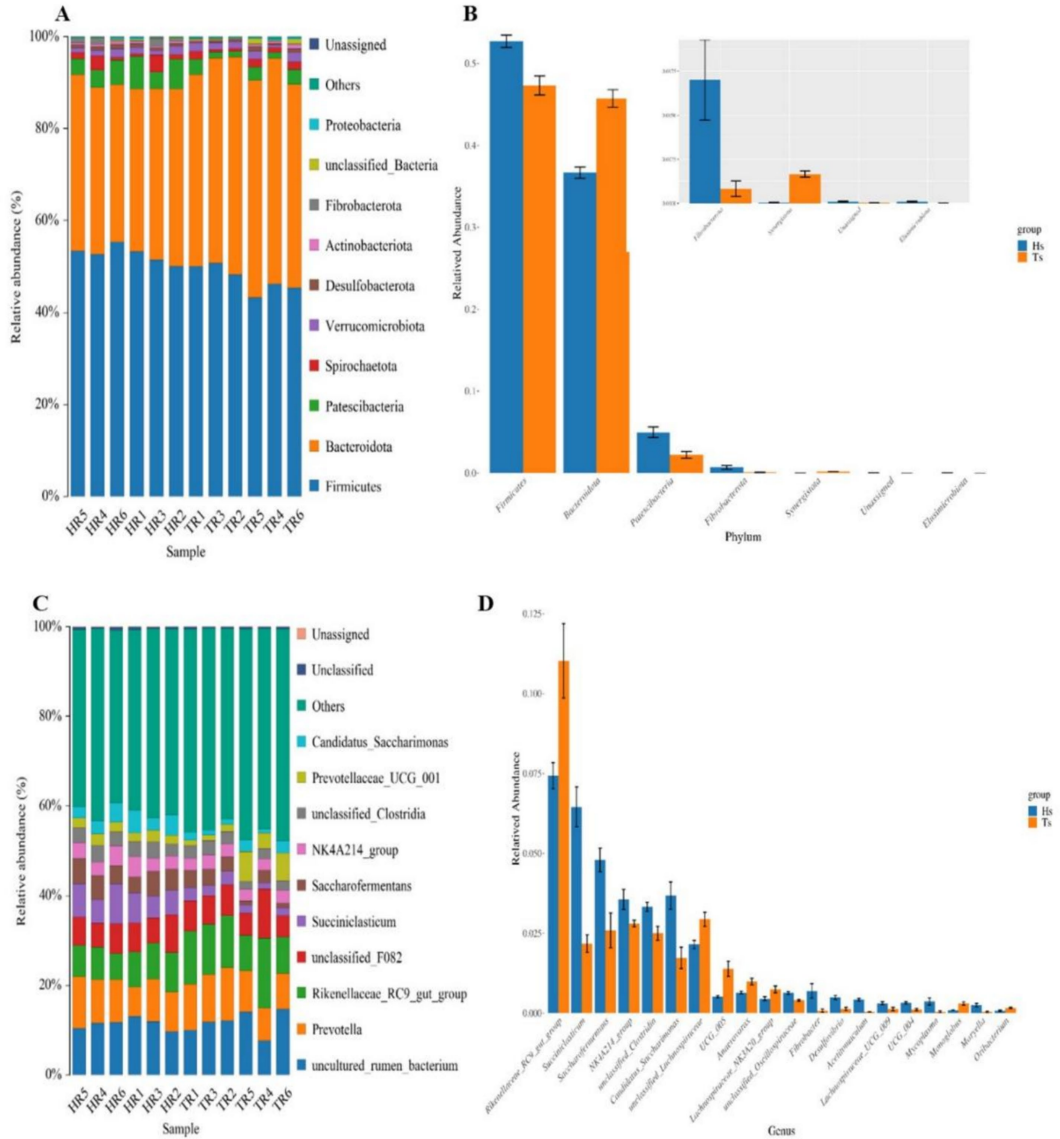
Analysis of the microbiological composition of Tibetan sheep and Hu sheep. **(A,C)** Microbial composition at the level of phylum and genus. **(B,D)** Analysis of species differences at phylum and genus level.

At the phylum level, Firmicutes, Bacteroidetes, Patescibacteria, Spirochaetota, and Verrucomicrobiota are the dominant phyla (Figure 3A). The abundances of Firmicutes and Patescibacteria are significantly greater (*p* < 0.05) in the Hu sheep than in the Tibetan sheep. In contrast, the abundance of Bacteroidetes is considerably greater (*p* < 0.05) in the Tibetan sheep than in the Hu sheep (Figure 3B). At the genus level, *uncultured_rumen_bacterium*, *Prevotella*, *Rikenellaceae_RC9_gut_group*, *unclassified_F082*, and *Succiniclasticum* are the dominant genera (Figure 3C), and in Tibetan sheep *Prevotella* and *Rikenellaceae_RC9_gut_group* abundance is significantly greater than that of Hu sheep (*p* < 0.05) (Figure 3D).

**Figure 4:**
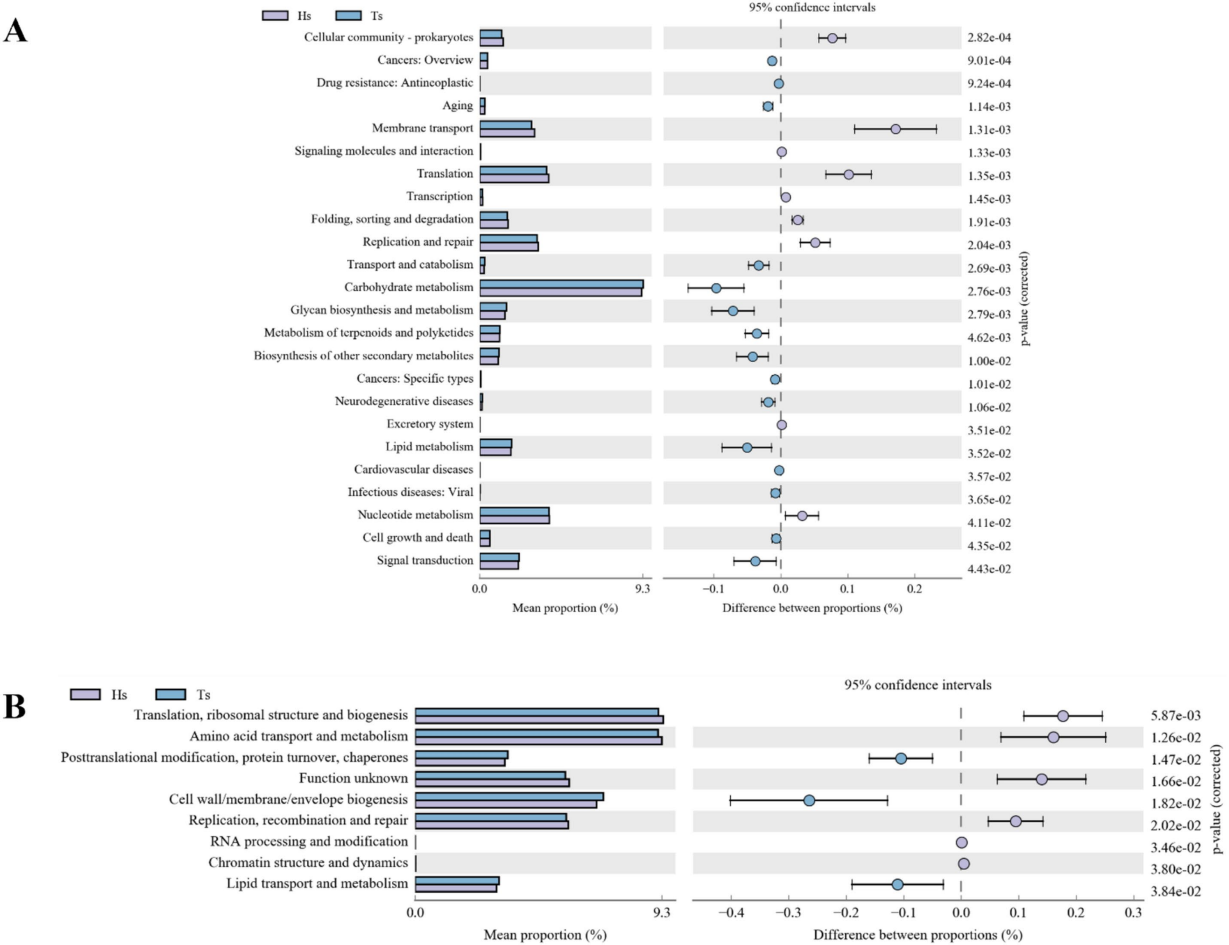
Intestinal microbial function analysis of Tibetan sheep and Hu sheep. **(A)** Kyoto Encyclopedia of Genes and Genomes (KEGG) functional analysis. **(B)** Clusters of Orthologous Groups of proteins (COG) functional analysis.

Ruminal differential microbial KEGG functional enrichment reveals that Membrane transport, Translation, Replication, and repair are significantly greater in Hu sheep compared to Tibetan sheep, while carbohydrate metabolism, glycan biosynthesis and metabolism, biosynthesis of other secondary metabolites, lipid metabolism, and signal transduction are significantly higher in Tibetan sheep than in Hu sheep (Figure 4A). COG functional analysis reveals that posttranslational modification, protein turnover, chaperones, and cell wall/membrane/envelope biogenesis functions were all significantly higher in Tibetan sheep than in Hu sheep, whereas in the rumen of Hu sheep, the ribosomal structure and biogenesis and amino acid transport and metabolism functions are greater in Hu sheep than in Tibetan sheep (Figure 4B).

### 3.3 Rumen microbial metabolomics assay

**Figure 5:**
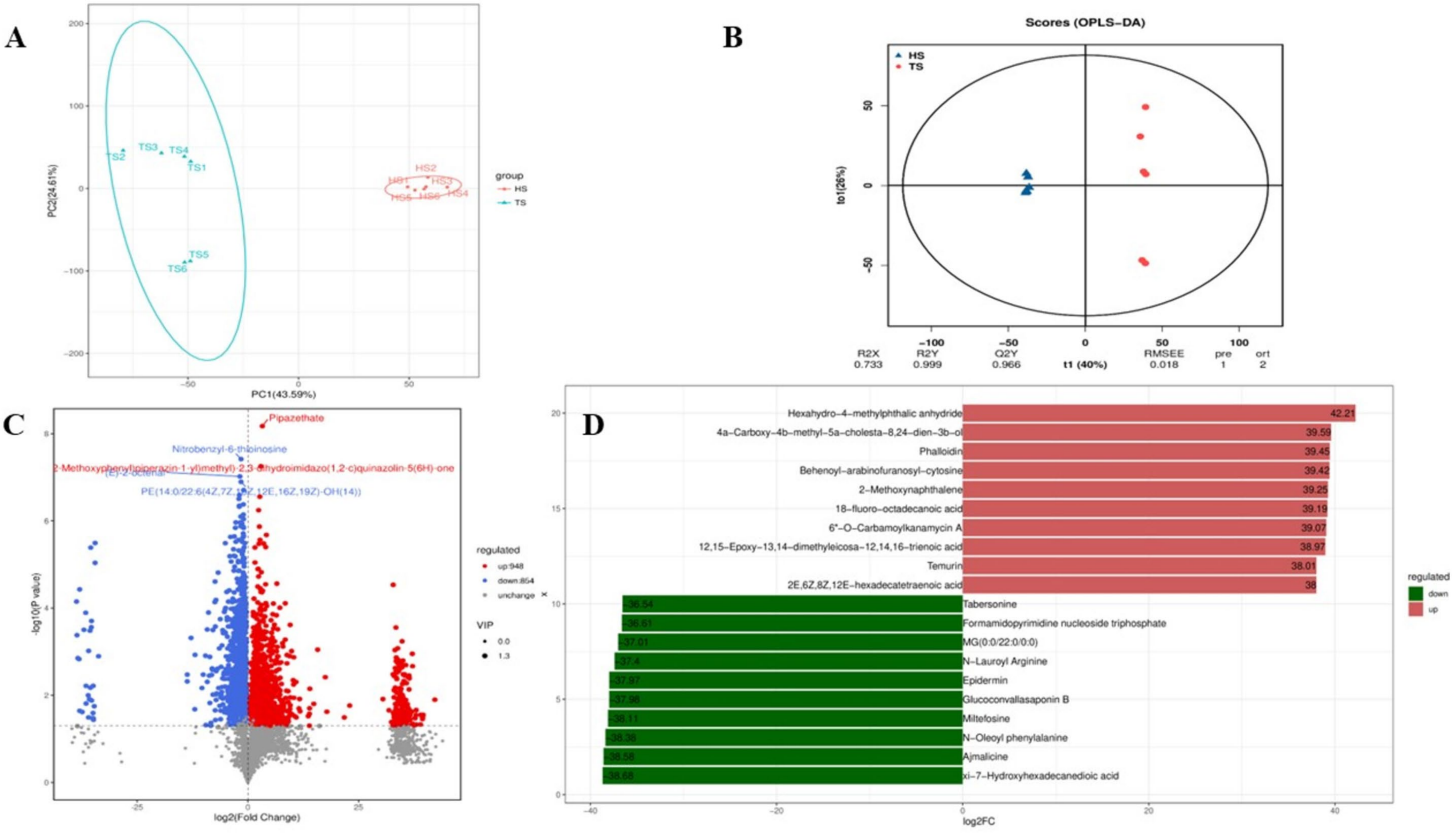
Quality control diagram of rumen microbial metabolome data of Tibetan sheep and Hu sheep. **(A)** Scatter plot of principal component analysis (PCA)-score showing the similarity of the rumen metabolites of Tibetan sheep and Hu sheep based on UniFrac distance, principal components (PCs) 1 and 2 explained 43.59 and 24.61% of the variance, respectively. **(B)** Orthogonal projections to late structures discriminant analysis (OPLS-DA) based on operational taxonomic units (OTUs) of rumen metabolites in Hu sheep and Tibetan sheep. **(C)** Volcanic maps of differential metabolites in Tibetan sheep and Hu sheep. **(D)** Column chart of positive ion difference multiples.

Analysis of rumen microbiota metabolites in Tibetan sheep and Hu sheep yielded a total of 959,773 pairs of reads. After double-ended reads quality control and splicing, a total of 877,838 clean reads are generated, with at least 72,663 clean reads produced per sample and an average of 73,153 clean reads produced. A total of 3,982 metabolites are identified. Principal component analysis (PCA) can reduce high-dimensional data to two-dimensional or three-dimensional space, making it easier to visualize and analyze the data. It can also identify the most representative information in the data, providing a visual understanding of its distribution characteristics and differences. The PCA results of this study reveal significant differences in the rumen microbial species composition between the Tibetan sheep and Hu sheep groups, with good intragroup reproducibility (Figure 5A). Orthogonal projections to late structures discriminant analysis (OPLS-DA) can be used to screen differentially abundant metabolites effectively in metabolomics analysis. OPLS-DA can be used to obtain intergroup differential information better by establishing a model between metabolite expression levels and grouping relationships and screening differentially abundant metabolites that contribute significantly to distinguishing different groups. OPLS-DA reveals that the R2X, R2Y, and Q2 values are all close to 1, with Q2 > 0.9, further validating the reliability of the OPLS-DA model (Figure 5B). Further cluster analysis is conducted to obtain the number of OTUs in each sample. At the taxonomic level, a total of 26 phyla, 58 classes, 124 orders, 210 families, 368 genera, and 450 species are detected. Differentially abundant metabolites are analyzed using FC > 1, *p* < 0.05, and VIP > 1 as the screening criteria, and a total of 1,802 differentially abundant metabolites are identified, of which 948 are upregulated and 854 are downregulated (Figure 5C). Further screening of the top 10 up- and downregulated metabolites with multiplicative differences reveal that phalloidin, hexahydro-4-methylphthalic anhydride, 12,15-epoxy-13,14-dimethyleicosa-12,14,16-trienoic acid, 2-methoxynaphthalene, 18-fluoro-octadecanoic acid, 6”-O-carbamoylkanamycin A, and Temurin are significantly upregulated in the rumen of Tibetan sheep and very low in Hu sheep, these metabolites play an important role in lipid metabolism, and can also promote bile secretion, inhibit cancer cell proliferation, and participate in immune regulation. Tabersonine, *N*-lauroyl arginine, epidermin, glucoconvallasaponin B, miltefosine, *N*-oleoyl phenylalanine, and ajmalicine are significantly upregulated in the rumen of Hu sheep, which are involved in the regulation of inflammation to ensure normal growth and development of Hu sheep (Figure 5D).

**Figure 6:**
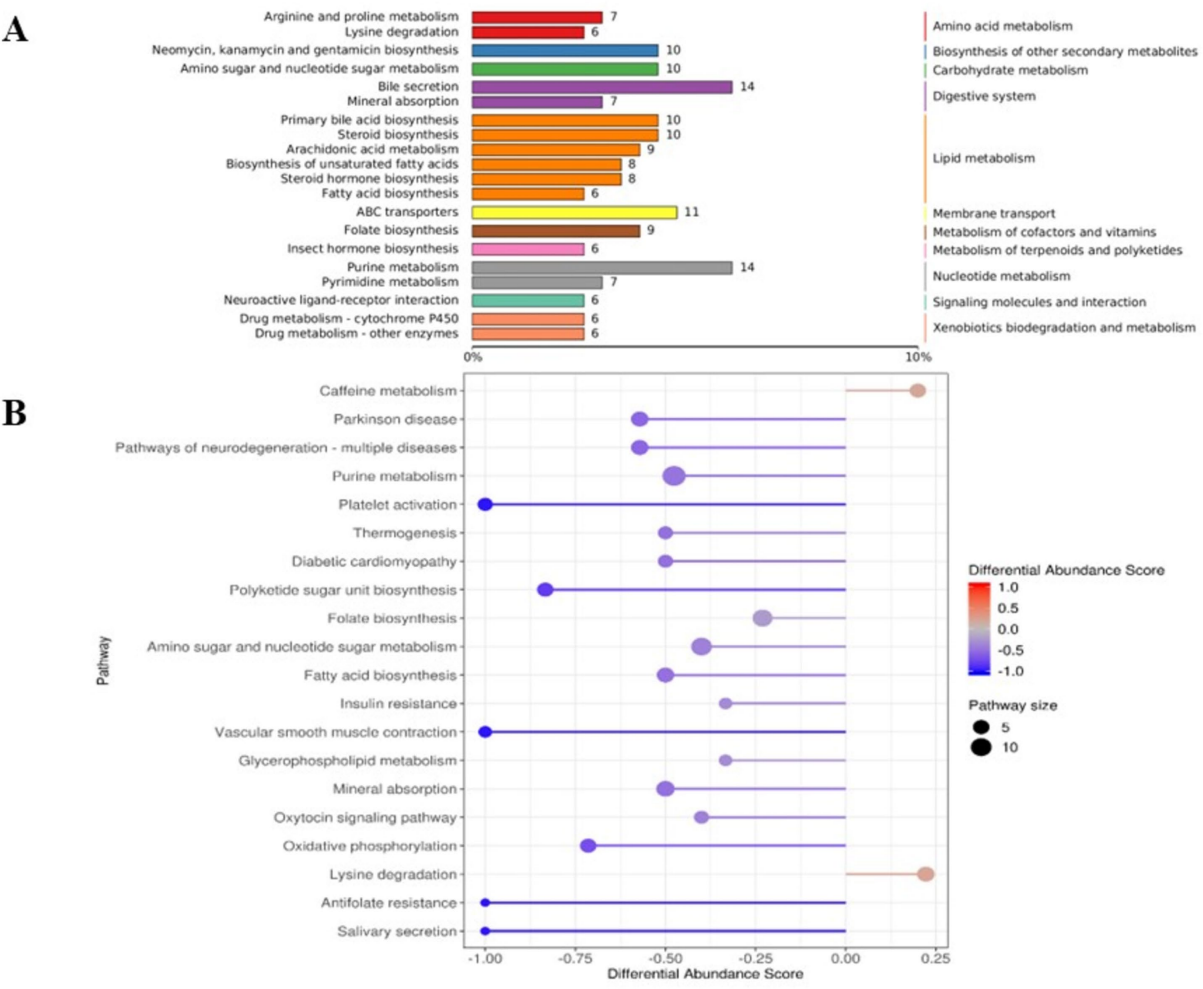
The Kyoto Encyclopedia of Genes and Genomes (KEGG) functional analysis diagram of differential metabolites in rumen microbiomes of Tibetan sheep and Hu sheep. **(A)** Differential metabolite KEGG pathway classification annotation. **(B)** Differential metabolite differential abundance score diagram.

The results of KEGG functional annotation and enrichment of different metabolites in the rumens of Tibetan sheep and Hu sheep are shown in Figure 6, and the different metabolites are enriched mainly in the lipid metabolism, digestive system, nucleotide metabolism, and amino acid metabolism pathways. Among them, the upregulated differentially abundant metabolites are enriched mainly in the caffeine metabolism and lysine degradation pathways, and the downregulated differentially abundant metabolites are enriched in a total of 18 metabolic pathways, with significant enrichment in the purine metabolism, amino sugar, and nucleotide sugar metabolism, folate biosynthesis, fatty acid biosynthesis, and mineral absorption pathways.

### 3.4 Rumen VFAs concentration and transporter gene expression

**Figure 7:**
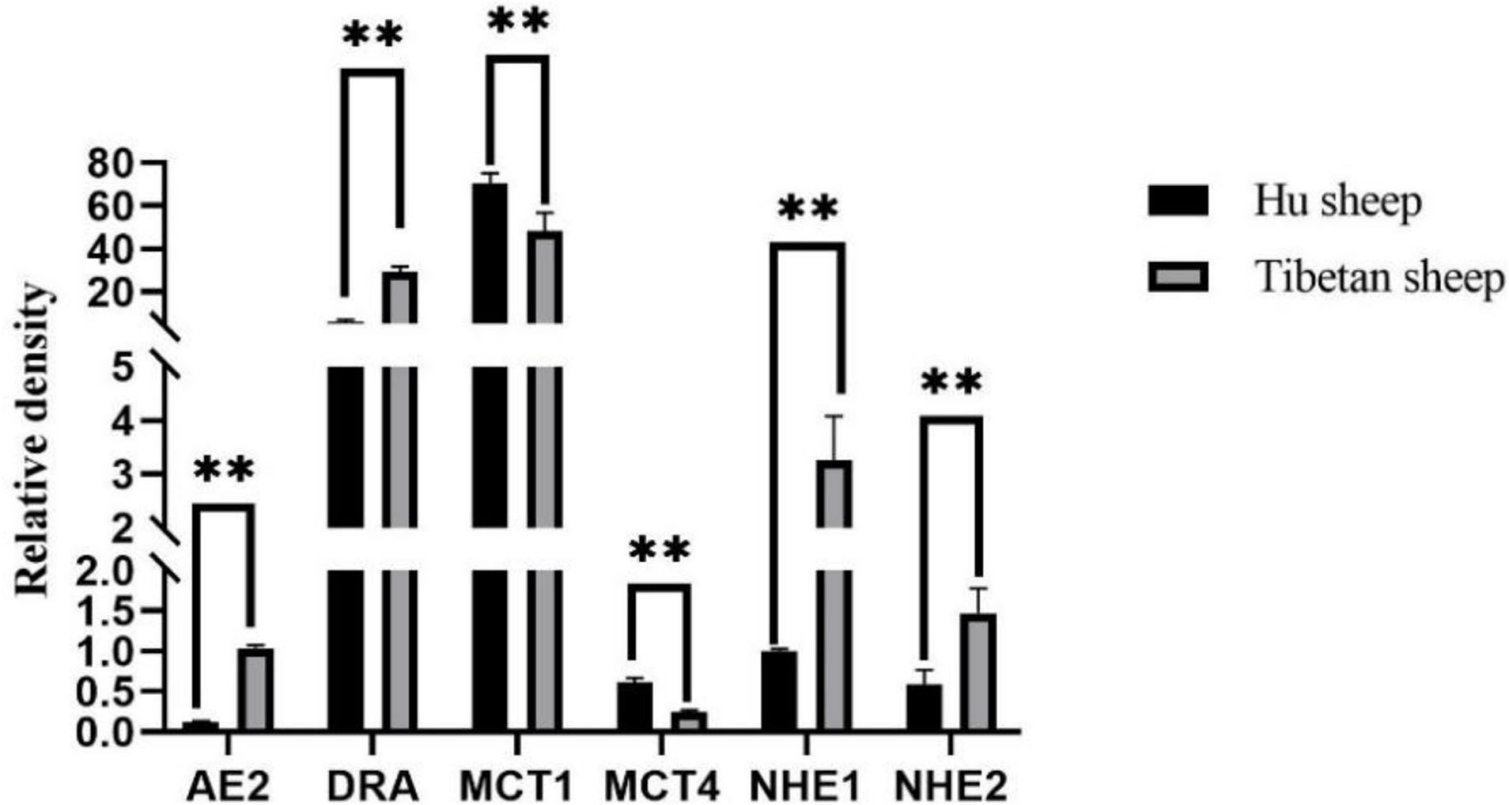
Rumen volatile fatty acids (VFAs) transport gene expression results of Tibetan sheep and Hu sheep. **Indicates highly significant difference (*p* < 0.01).

As shown in Table 2, there is a significant difference in the concentration of rumen VFAs between Tibetan sheep and Hu sheep, in which the total rumen VFAs concentration of Hu sheep is significantly greater than that of Tibetan sheep (*p* < 0.05). The concentrations of acetate, propionate, butyrate, and valerate in the Hu sheep rumen are greater, and the differences are significant (*p* < 0.05) for acetate, propionate, and valerate. The concentrations of isobutyrate and isovalerate acids are significantly greater (*p* < 0.05) in Tibetan sheep than in Hu sheep, and the Acetate/Propionate (A/P) values are significantly greater (*p* < 0.05) in Tibetan sheep than in Hu sheep. The results of the relative expression measurements of rumen VFAs transporter genes (*AE2*, *DRA*, *MCT1*, *MCT4*, *NHE1*, and *NHE2*) are shown in Figure 7. The expression levels of *AE2*, *DRA*, *NHE1*, and *NHE2* are significantly greater in Tibetan sheep than in Hu sheep (*p* < 0.05), whereas the expression levels of *MCT1* and *MCT4* are significantly greater in Hu sheep than in Tibetan sheep (*p* < 0.05).

**Table 2:** Contents of volatile fatty acids (VFAs) in the rumen of Tibetan sheep and Hu sheep.

**Place**	**Project**	**Hu sheep**	**Tibetan sheep**	***p-*value**
Rumen	Acetate/(mmol/L)	30.191 ± 1.682	21.939 ± 0.898	0.002
	Propionate/(mmol/L)	6.118 ± 0.233	3.392 ± 0.214	0.000
	Isobutyrate/(mmol/L)	0.198 ± 0.027	0.436 ± 0.049	0.002
	Butyrate/(mmol/L)	2.412 ± 0.332	1.903 ± 0.025	0.057
	Isovalerate/(mmol/L)	0.239 ± 0.061	0.612 ± 0.035	0.001
	Valerate/(mmol/L)	1.162 ± 0.027	0.643 ± 0.015	0.000
	VFAs/(mmol/L)	40.321 ± 1.319	28.926 ± 0.869	0.000
	A/P/(mmol/L)	4.9436 ± 0.415	6.4924 ± 0.628	0.024

### 3.5 Analysis of rumen microbe–metabolite interactions

**Figure 8:**
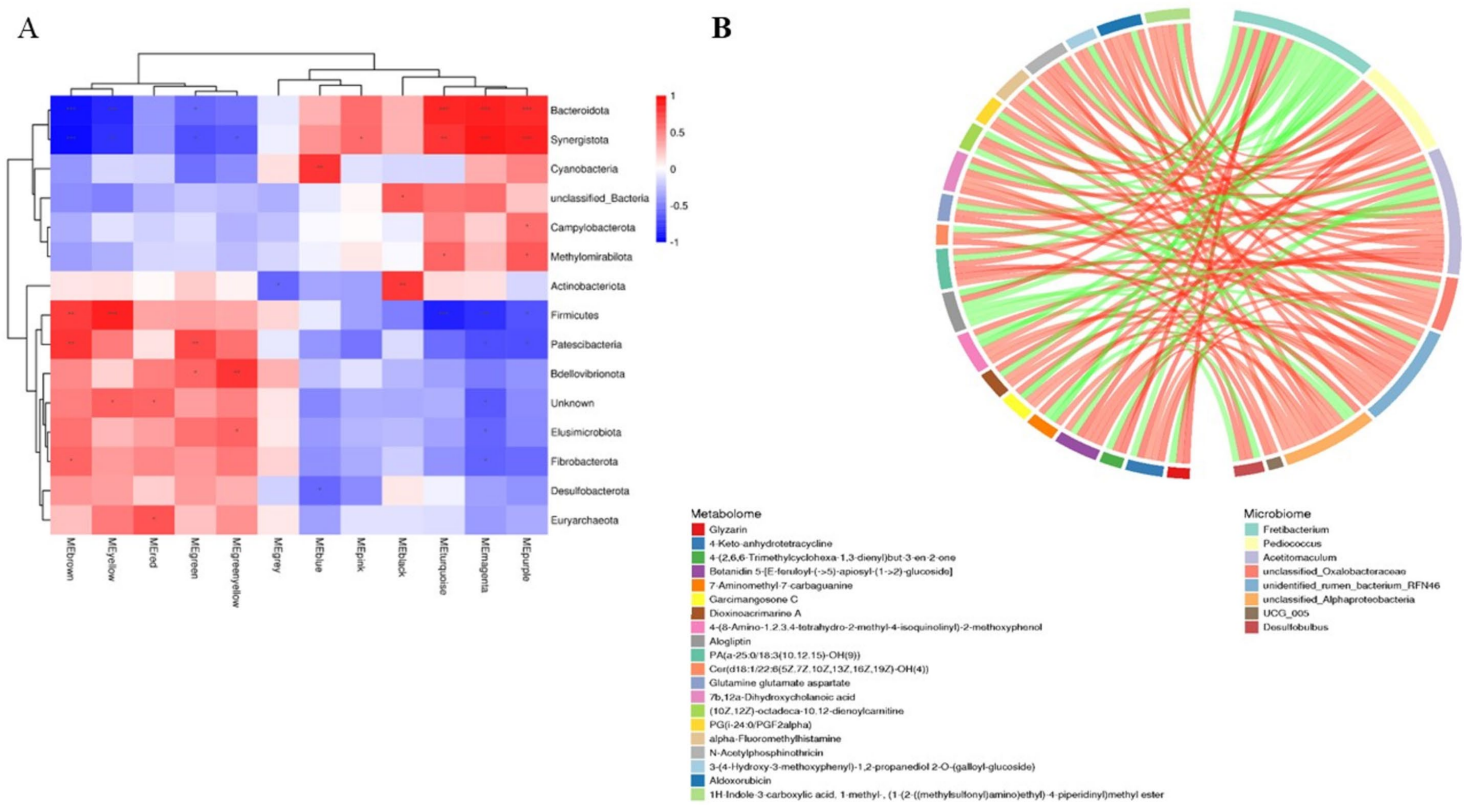
Correlation analysis of rumen microbiomes and metabolites in Tibetan sheep and Hu sheep. **(A)** Microbiomes and metabolites correlation heat map. **(B)** Microbiomes and metabolites correlation chord diagram. *p < 0.05, **p < 0.01, ***p < 0.001.

Analysis of rumen microbiota and its metabolites reveals a certain correlation between the two (Supplementary Figure S3). Further correlation analysis was conducted, and the heatmap of microbiota and metabolites at the phylum level (Figure 8A) revealed that microorganisms are correlated with 12 metabolite modules, of which Bacteroidota and Synergistota are highly significantly positively correlated with three metabolite modules, MEturquoise, MEmagenta, and MEpurple (*p* < 0.01); Firmicutes is highly significantly positively correlated with MEbrown and MEyellow; two modules are highly significantly positively correlated (*p* < 0.01); and Patescibacteria is highly significantly positively correlated (*p* < 0.01) with the MEbrown and MEgreen modules, whereas Bacteroidetes and Synergistota are highly significantly negatively correlated (*p* < 0.01) with the MEbrown, MEyellow and MEgreen modules. Further correlation and chord plots of microorganisms and metabolites at the genus level revealed that rumen microorganisms and metabolites are also inextricably linked. *Fretibacterium* is significantly negatively correlated (*p* < 0.05) with Glutamine glutamate aspartate, Aldoxorubicin, Gyrocyanin, and *α*-Fluoromethylhistamine, whereas Pediococcus is significantly positively correlated (*p* < 0.01) with glutamine, glutamate, and aspartate (*p* < 0.01). *Acetitomaculum* and *Pediococcus* are strongly positively correlated with Aldoxorubicin, Gyrocyanin, and α-Fluoromethylhistamine (*p* < 0.01) (Figure 8B).

### 3.6 Analysis of rumen microbe-VFAs and transporter gene–epithelial morphology interactions

**Figure 9:**
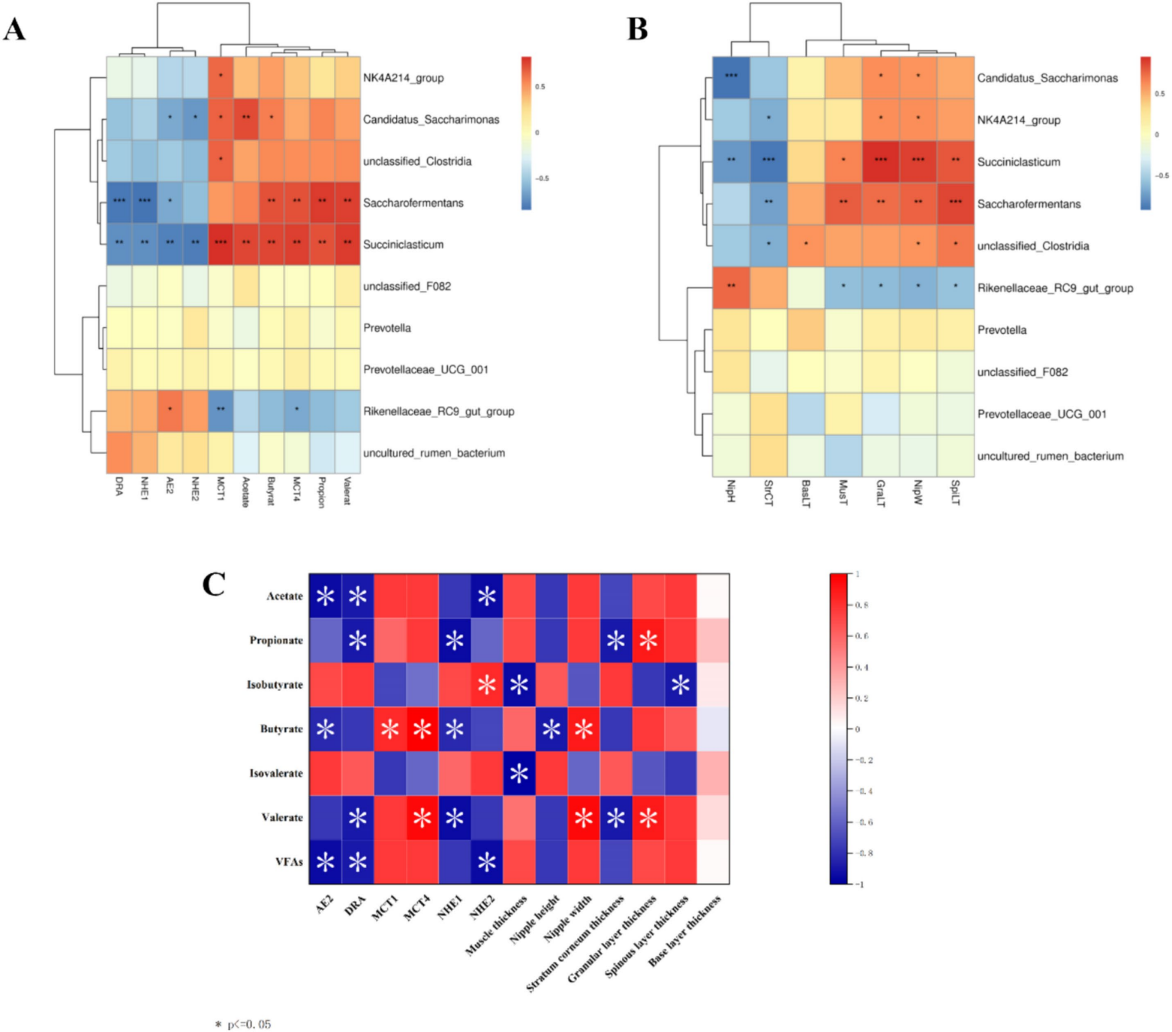
Correlation heat map between Tibetan sheep and Hu sheep. **(A)** Heat map of correlation between horizontal rumen microbiomes and VFAs and transport genes. **(B)** Heat map of morphological correlation between rumen microbiomes and rumen epithelium. **(C)** Heat map of correlation between rumen VFAs and transport genes.

Interaction analysis of the top 10 rumen microorganisms at the genus level with VFAs and their transporter genes and rumen epithelial morphology reveals that *Rikenellaceae_RC9_gut_group* is significantly positively correlated (*p* < 0.05) with nipple height and the *AE2* gene and negatively correlated (*p* < 0.05) with the *MCT1* and *MCT4* genes and nipple width, *Succiniclasticum* and *Saccharofermentans* are strongly positively correlated with propionate, butyrate, valerate, and *MCT4* genes, as well as with nipple width, granular layer thickness, and sphenoid layer thickness (*p* < 0.01) and *Succiniclasticum* is also highly significantly positively correlated with acetate and *MCT1* genes (*p* < 0.01), and highly significantly negatively correlated with the *AE2*, *DRA*, *NHE1*, and *NHE2* genes, as well as with nipple height and cuticle thickness, which are highly significantly negatively correlated (*p* < 0.01), (Figures 9A,B). Spearman correlation analysis of rumen VFAs, transporter genes, and their rumen epithelial micromorphology in Tibetan sheep and Hu sheep. As shown in Figure 9C, the *AE2*, *DRA*, and *NHE2* genes are significantly and positively correlated (*p* < 0.05) with the contents of acetate and total acids, and the *DRA* and *NHE1* genes and their rumen epithelial cuticle thickness are significantly and positively correlated (*p* < 0.05) with the contents of propionic acid and valerates. The *AE2* and *NHE1* genes and the nipple height are significantly positively correlated with the butyrate content (*p* < 0.05), whereas the *MCT1* and *MCT4* genes and the nipple width are significantly negatively correlated with the butyrate content (*p* < 0.05). These findings suggest that rumen transporter genes and the rumen epithelial structure are inextricably linked to the production and transporter uptake of VFAs.

### 3.7 Weighted correlation network analysis (WGCNA) of rumen microbial metabolite-VFAs transporter genes

**Figure 10:**
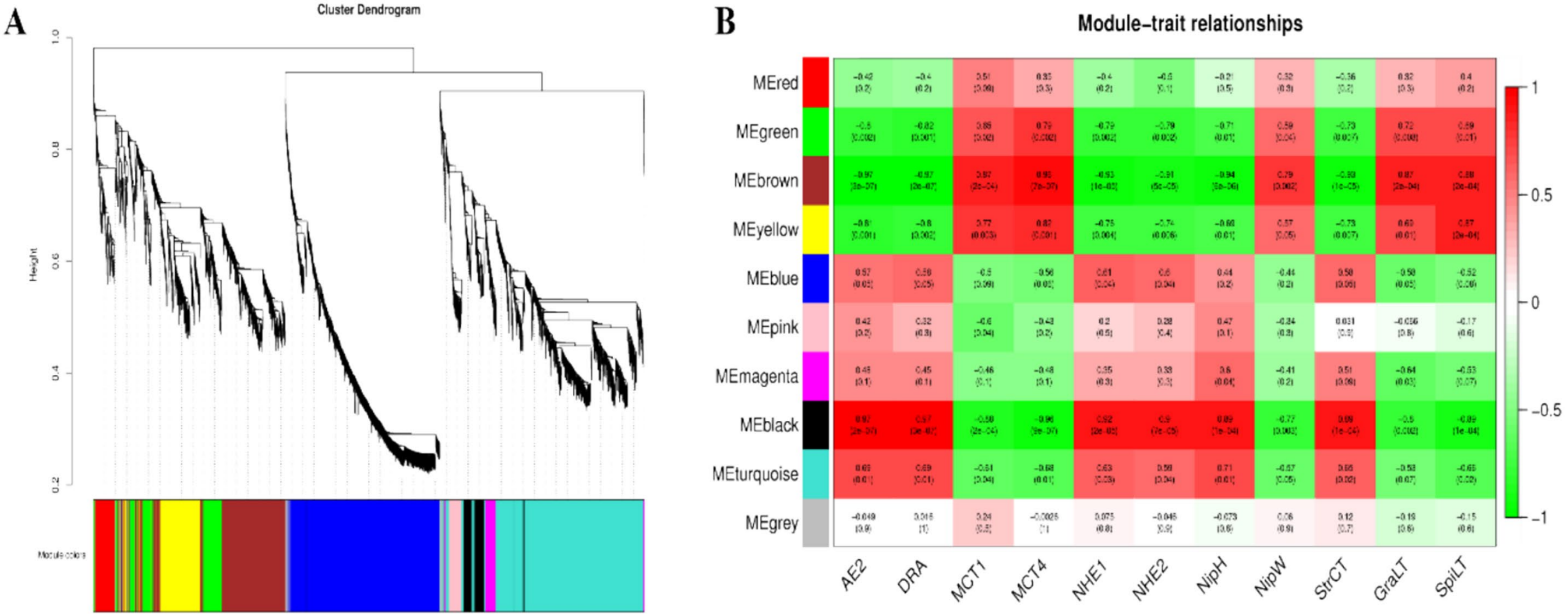
The results of WGCNA. **(A)** Cluster dendrogram of rumen metabolites in Tibetan sheep and Hu sheep. Each branch in the figure represents one metabolite, and each color represents one coexpression module. **(B)** Correlation between the rumen metabolite modules, transport genes and rumen epithelial morphology in Tibetan sheep and Hu sheep.

Based on the results of the rumen microbial metabolomics analysis, further WGCNA of 1,802 differentially abundant metabolites reveal that 10 modules are differentially correlated with rumen epithelial morphology and transporter genes related to VFAs in Tibetan sheep and Hu sheep (Figure 10). There is a strong correlation between the five modules, MEgreen, MEbrown, MEyellow, MEblack, and MEturquoise, and rumen epithelial morphology and VFAs-related transporter genes. The MEgreen, MEbrown, and MEyellow modules are significantly and positively correlated with the *MCT1* and *MCT4* genes and the thicknesses of the granular and sphenoid layers. The MEblack and MEturquoise modules are positively associated with the *AE2*, *DRA*, *NHE1*, and *NHE2* genes, nipple height, and cuticle thickness, whereas they are negatively correlated with the *MCT1* and *MCT4* genes. Further analysis of the metabolites with the top 10 kME values in the five modules revealed that the metabolites in the MEgreen, MEbrown, and MEyellow modules are enriched in the biosynthesis of unsaturated fatty acids and Fructose and mannose metabolisms such as downregulated metabolites such as alginic acid and linoleic acid; In contrast, in the MEblack and MEturquoise modules, some upregulated metabolites, such as 3α7α,12α-trihydroxy-5β-cholestanoate, 5-hydroxyectoine, and dihydrotestosterone, which are found in Tibetan sheep, were significantly more abundant than those in Hu sheep and are enriched mainly in the pathways of primary bile acid biosynthesis, glycine, serine and threonine metabolism, and steroid hormone biosynthesis.

## 4 Discussion

This study aimed to analyze the breed differences in the rumen epithelial micromorphology, microbiome, metabolome, and expression levels of VFAs and their transporter genes between Tibetan sheep and Hu sheep under the same feeding conditions and to investigate the mechanism of plateau adaptation in Tibetan sheep on the basis of the rumen microbiome and its metabolome. The rumen is one of the most important sites for digestion, metabolism, and nutrient absorption in ruminants, and previous studies have shown that nutrient absorption across the rumen epithelium is mainly dependent on the degree of keratinization of the cuticle cells (Baldwin and Jesse, 1992). In this study, the thickness of the rumen nipple and cuticle is greater in Tibetan sheep than in Hu sheep because of their greater digestibility of alpine forage of coarse quality and high fiber content, which require strong rumen contraction and diastole to achieve mechanical digestion. The sphenoid layer is the leading site of VFA metabolism in rumen epithelial tissue. Compared with Tibetan sheep, Hu sheep need to metabolize more VFAs for energy to adapt to the plateau environment. The lower thickness of the rumen sphenoid layer in Tibetan sheep indicates that Tibetan sheep inhibit further metabolism of VFAs, and store them for their energy supply under conditions of hypoxia and severe nutritional deficiency. The height of the rumen nipple in Tibetan sheep is greater than that in Hu sheep when stimulated by plateau-wilting grasses. Therefore, a higher rumen nipple is a trait and strategy for the digestion and utilization of alpine pastures in Tibetan sheep and their adaptation to the plateau environment.

Rumen microorganisms play an important role in the digestive metabolism of proteins, sugars, and fats in ruminants. In this study, Firmicutes and Patescibacteria are more abundant in the rumen of Hu sheep at the phylum level, and Firmicutes promote fiber degradation, growth, and development (Lin et al., 2018). In contrast, the abundance of Bacteroidetes is greater in Tibetan sheep and has been observed to be much lower than Firmicutes in the rumen microbiota of low-altitude ruminants (Kim et al., 2011) and goats from semiarid regions (Cunha et al., 2011). Bacteroidetes can efficiently catabolize dietary proteins and carbohydrates into VFAs (Huo et al., 2014) and promote increased rumen growth and volume. The large number of bacteria in Bacteroidetes in plateau ruminants highlights the vital role of these bacteria, which may be beneficial in providing nutrients to the host by degrading the limited resources in the Qinghai-Tibet Plateau (QTP) (Liu et al., 2020). Furthermore, Bacteroidetes play an important role in nutrient uptake and utilization and host disease immunity (Zhang et al., 2018), thus playing a more important role than Firmicutes in the adaptation of animals to high-altitude environments. These findings suggest that Tibetan sheep have a stable energy utilization mechanism when the pasture supply is insufficient at high altitudes, which is important for maintaining homeostasis in the rumen and adapting to harsh environments (Jami et al., 2014). At the genus level, *Rikenellaceae_RC9_gut_group*, which degrades polysaccharides of plant origin (Peng et al., 2015), is significantly more abundant (*p* < 0.05) in the rumen of Tibetan sheep than in that of Hu sheep. In addition, *Prevotella* is also a dominant genus in Tibetan sheep, and *Prevotella* plays a role in the degradation and utilization of plant non-cellulosic polysaccharides, proteins, starches, and xylans (Liu et al., 2019). A high abundance of *Prevotella* promotes forage fermentation, resulting in high concentrations of VFAs (Shi et al., 2014), which facilitate the ability of rumen-fermented forage to provide more energy to the organism. Furthermore, KEGG functional enrichment analysis reveals that Carbohydrate metabolism, Glycan biosynthesis and metabolism, and Lipid metabolism are significantly enhanced in Tibetan sheep, which are able to satisfy their energy needs in the alpine hypoxic environment (Fan et al., 2021), whereas functions such as Membrane transport, which can mediate interactions between the intestinal microbiota and host cells (Konishi et al., 2015), are significantly elevated in the rumen of Hu sheep. The COG results also reveal that posttranslational modification, protein turnover, chaperones, and cell wall/membrane/envelope biogenesis are increased in Tibetan sheep. Protein turnover breaks down proteins into amino acids during food digestion for growth and development and is also involved in cellular signaling to adapt to environmental changes (Gonzalez and Cullen, 2022). The above results suggest that, compared with Hu sheep under the same feeding conditions, Tibetan sheep may have more powerful energy metabolism mechanisms to adapt to the Tibetan Plateau environment than do Hu sheep because their genetic factors lead to increased abundances of Bacteroidetes and *Prevotella*, effective fermentation and utilization of forage, and further enhancement of carbohydrate and lipid metabolism.

Metabolomics plays an important role in understanding the physiological and biochemical state of animals (Xue et al., 2020).In the present study, some differences in rumen are detected metabolites between Tibetan sheep and Hu sheep. Tabersonine, Epidermin, and Glucoconvallasaponin B levels are increased in Hu sheep, whereas metabolites such as phalloidin and 12,15-epoxy-13,14-dimethyleicosa-12,14,16-trienoic acid are increased in the rumens of Tibetan sheep. Phalloidin promotes bile secretion(Rahman and Coleman, 1986), facilitates fat digestion, and inhibits bacterial proliferation, and 12,15-epoxy-13,14-dimethyleicosa-12,14,16-trienoic acid is a furan fatty acid derivative that has been shown to inhibit cancer cell proliferation and block cell recovery. These results suggest that differentially abundant metabolites play important roles in fatty acid catabolism and the intestinal immune barrier in Tibetan sheep, but the specific regulatory mechanisms need to be further verified. Further analysis of the KEGG functional enrichment of the differentially abundant metabolites reveals that the differentially abundant metabolites of Tibetan sheep and Hu sheep are enriched mainly in the Lipid metabolism, Digestive system, nucleotide metabolism, and amino acid metabolism pathways. Among them, lipid metabolism plays an important role in energy metabolism as well as in various aspects of biofilm structure, signaling and other functions, and disorders of lipid metabolism can lead to a variety of diseases (Petrenko et al., 2023), and its enrich metabolites, such as cholesterol, the expression of which is upregulated in the rumen of Tibetan sheep, are precursors for the synthesis of a variety of hormones, such as steroid hormones and glucocorticosteroids, which are related to immunity and energy metabolism (Cortes et al., 2014). In addition, caffeine metabolism and Lysine degradation are upregulated in Tibetan sheep, and its upregulated metabolites, Theophylline and Xanthine, are associated with the regulation of inflammation (Rabe and Dent, 1998; Yagi et al., 2022). Pyruvate and alanine produced by Lysine degradation can further participate in energy metabolism and amino acid metabolism processes. Purine metabolism, amino sugar and nucleotide sugar metabolism, and fatty acid biosynthesis are significantly enriched in Hu sheep and participate in various biological processes to ensure normal growth and development. The above analyses reveal significant differences in rumen microbial metabolites and their enrichment pathways between Tibetan sheep and Hu sheep under the same feeding environment, which is due mainly to breed differences, with Tibetan sheep producing more metabolites related to organismal energy provisioning and immunomodulation than Hu sheep do.

Under the regulation of rumen microorganisms and their metabolites, VFAs also change accordingly. Energy supply is the most important role of VFAs in ruminants, and the rumen acetate content accounts for 70 ~ 75% of the total VFA content. Increasing the level of acetate in the rumen not only alters the fermentation pattern in the rumen, improving the efficiency of energy metabolism and reducing energy losses in ruminants, but also improves the utilization of nitrogen. Propionate is largely converted to glucose, and in sheep, the glucose produced from propionate provides 30 ~ 50% of the requirement. The concentrations of both acetate and propionate in the rumen of Hu sheep are significantly higher than those in the rumen of Tibetan sheep, indicating that the rumen of Hu sheep has a greater fermentation capacity for forage than that of Tibetan sheep. Most of the butyrate in the rumen is converted by rumen epithelial cells into ketones or CO_2_, which are byproducts of fatty acid oxidation (McGarry and Foster, 1980). The A/*p* value is related to the efficiency of forage energy utilization; the lower the ratio is, the greater the energy utilization efficiency of the organism (Liu et al., 2019). The A/p value of Hu sheep is significantly lower than that of Tibetan sheep, which indicates that Hu sheep have a higher utilization rate of forage and can produce more VFAs; however, in plateau environments, the metabolism of energy substances in Tibetan sheep is greater than that in Hu sheep. VFAs, as important products of rumen fermentation, are absorbed and transported in the rumen epithelium by passive diffusion and anion exchange (del Bianco Benedeti et al., 2018), and the substances that play these roles are mainly transporter genes, such as the *AE2*, *DRA*, *NHE*, and *MCT* genes. Both *AE2* and *DRA* encode anion-exchange proteins that exchange VFA^−^ for HCO_3_^−^ and play important roles in regulating the stability of the internal environment. Zhang et al. reported that *AE2* may be involved in maintaining the intracellular pH balance during the transport of VFAs (Zhang et al., 2023), which indirectly affects the transport and metabolism of VFAs, and high expression of *AE2* may indicate that the cellular capacity for bicarbonate transport is increased, which contributes to maintaining the stability of the intracellular environment and supports the further metabolism of VFAs. Bilk et al. (2005) reported that the rumen epithelium exchanges HCO_3_^−^ and Cl^−^ via *AE2*, *DRA,* and *PAT1* and is associated with the uptake and transport of VFAs in this process. *NHE* is a membrane transporter of Na^+^ and H^+^ that transports VFAs mainly by passive diffusion (Müller et al., 2002), and it has been demonstrated that *NHE1* and *NHE2*, among others, are expressed in the rumen epithelium and that their activities can influence the uptake of VFAs by the rumen epithelium. Yang et al. (2012) reported that the expression and activity of rumen epithelial *NHEs* were increased when goats were fed highly fermented diets, and in the present study, the expression of the above four genes in the rumen of Tibetan sheep was significantly greater than that in the rumen of Hu sheep, indicating that the absorption and metabolism of VFAs by Tibetan sheep in the plateau environment is stronger than that of Hu sheep and that the content of VFAs is lower than that of Hu sheep. Monocarboxylic acid transporter (MCT) proteins, which are distributed at the plasma membrane surface of cells in the basal layer of the rumen epithelium, are involved in regulating the efflux of H^+^ lactate and ketone bodies and maintaining intercellular pH homeostasis (Kirat et al., 2006). The results of the present study revealed that the expression of *MCT1* and *MCT4* in the rumen epithelium of Hu sheep was significantly greater than that in the rumen epithelium of Tibetan sheep. Pérez de Heredia et al. (2010) reported that hypoxia can upregulate the expression of *MCT1* and *MCT4* in human adipocytes and that the impression of *MCT4*, similar to other enzymes involved in glucose catabolism, is increased upregulated under hypoxic stimulation through the regulatory mechanism of HIF-1α, and its upregulated expression enables the rapid efflux of lactic acid from the cell due to sugar catabolism during hypoxia, which is in line with the results of the present study. Therefore, efficient uptake and metabolism of VFAs constitute another characteristic and effective strategy for Tibetan sheep to cope with the harsh alpine environment.

On the basis of the above analyses, we identified a certain synergistic relationship between the rumen microbiome and metabolome and their fermentation functions. In this study, the modules that are significantly positively correlated with Bacteroidetes are mainly related to immune-related metabolites such as Nylidrin, Physalin E acetate, and syringic acid. Among them, syringic acid, which has a structural unit similar to that of lignin, acts as an antioxidant to reduce oxidative stress and exerts antioxidant and anti-inflammatory effects (Ferah Okkay et al., 2022). *Bacteroidota*, a dominant genus of bacteria in Tibetan sheep, has been reported to be associated with microbial dysbiosis and chronic intestinal inflammation (Parker et al., 2020), suggesting that *Bacteroidota* and these metabolites are collectively involved in the regulation of intestinal immunity in Tibetan sheep, thus ensuring their health. *Pediococcus* spp. strains have the potential to inhibit mycotoxin-producing molds as producers of potent antifungal metabolites (Fugaban et al., 2022), which are significantly and positively correlated with metabolites such as Glutamine glutamate aspartate, which is a major metabolic fuel for the small intestine and can improve hepatic energy metabolism (Qi et al., 2020). In addition, there was an association between the rumen microbiota flora, rumen epithelial micromorphology, and VFAs in Tibetan sheep. *Rikenellaceae_RC9_gut_group* is significantly and positively correlated with nipple height and the *AE2* gene (*p* < 0.05), while R*ikenellaceae_RC9_gut_group* may promote the digestion and absorption of carbohydrates in the intestinal tract (Berry et al., 2015) and inhibit the production of proinflammatory cytokines and other damaging factors (Bian et al., 2019), and *Rikenellaceae_RC9_gut_group* and *Succiniclasticum* may be involved in methane formation and VFAs production. In this study, *Rikenellaceae_RC9_gut_group* and *Succiniclasticum* as the dominant genera, increased the content of VFAs, indicating that Tibetan sheep regulate the digestion and absorption of VFAs through the upregulation of genes, such as *Rikenellaceae_RC9_gut_group*, to promote the growth of the rumen nipple and *AE2*. This, in turn, enhances energy metabolism and immune regulation in Tibetan sheep to ensure their growth and development. Therefore, rumen microbes and their metabolites synergistically regulate VFAs and rumen epithelial morphology to improve energy metabolism and reduce oxidative stress in Tibetan sheep in the same feeding environment.

**Figure 11:**
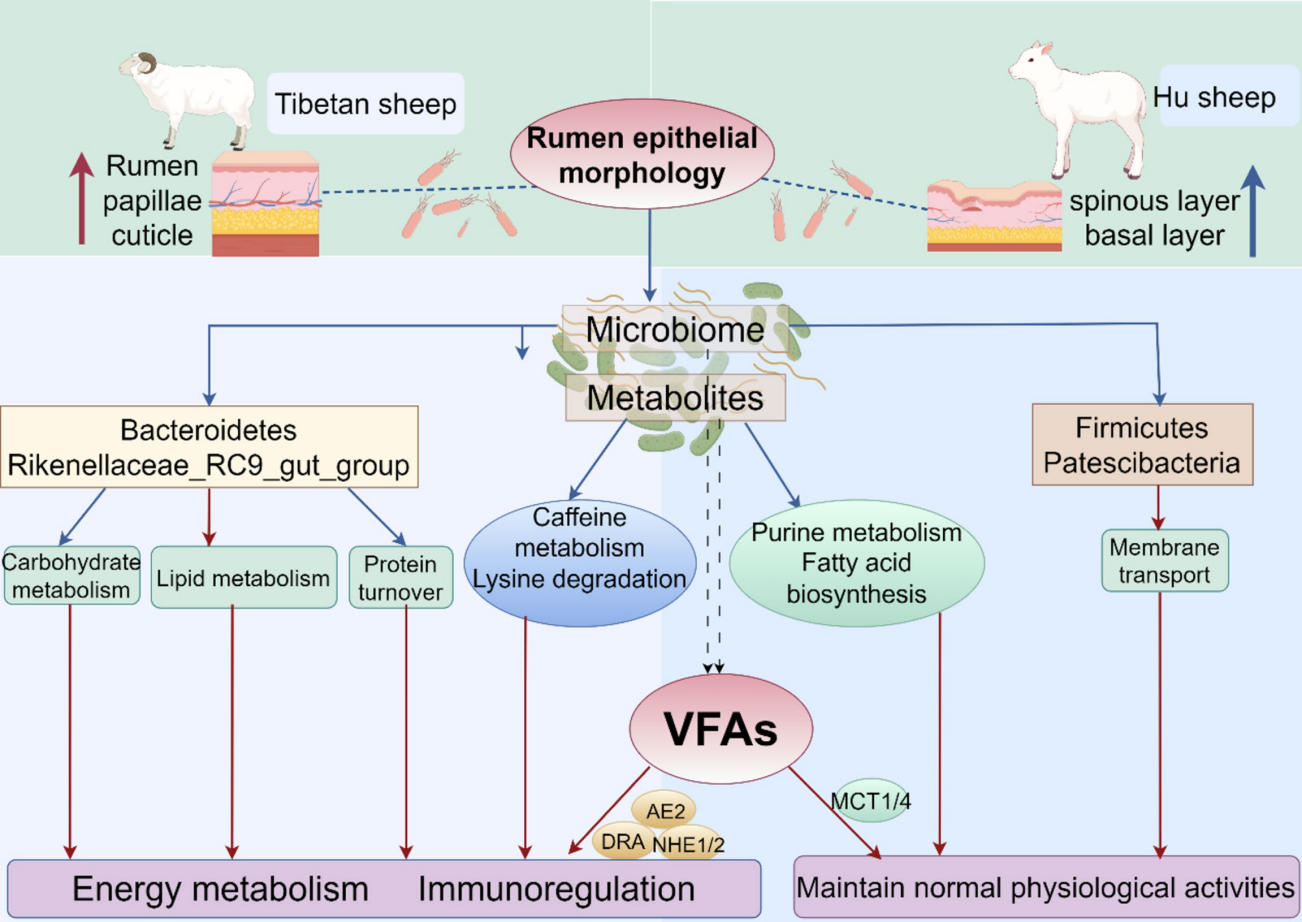
Modeling of rumen microorganisms and their fermentative metabolism in Tibetan sheep and Hu sheep.

Furthermore, WGCNA of rumen metabolites reveals that metabolite modules associated with highly expressed VFAs transporter genes and rumen epithelial micromorphology act mainly through saturated and unsaturated fatty acid biosynthesis and Fructose and mannose metabolism pathways to maintain normal physiological activities in Hu sheep. Several metabolites related to energy metabolism and immune barriers have been identified in Tibetan sheep, among which 3α,7α,12α-trihydroxy-5α-cholestanoate is an important precursor for bile acid synthesis(Mihalik et al., 2002). Bile acid promotes both the digestion and absorption of dietary lipids and acts as a hormone that activates specific receptors. The activation of these receptors can alter gene expression in a wide range of tissues, leading to changes not only in bile acid metabolism but also in glucose homeostasis, lipid and lipoprotein metabolism, energy expenditure, intestinal motility, and bacterial growth, and inflammation (de Aguiar et al., 2013). 5-Hydroxyectoine can bind to some 5-hydroxyectoine receptors on the surface of immune cells to regulate the immune system and participate in immune metabolism. In addition, many metabolites related to amino acids, such as Gly, Ser, and Thr, are involved in immune function, anti-inflammatory processes, and antioxidant responses, in addition to meeting the normal developmental and amino acid requirements of Tibetan sheep (Imenshahidi and Hossenzadeh, 2022). The interactions between the above metabolites and VFAs and epithelial morphology further provide insight into plateau adaptation in Tibetan sheep (Figure 11).

## 5 Conclusion

In the case of rough grass quality on the plateau, the high rumen nipple and thick cuticle of Tibetan sheep are the main differences in their adaptation to high-altitude environments compared to Hu sheep, Second, the rumen microbiota of Tibetan sheep has increased abundances of Bacteroidetes, *Prevotella* and *Rikenellaceae_RC9_gut_group*, which efficiently ferment and utilize forage and absorb the metabolize VFAs, promote the expression of VFAs transporter genes, and improve carbohydrate, amino acid and lipid metabolism, thus giving Tibetan sheep a more powerful energy metabolism mechanism to adapt to the environment. In addition, Tibetan sheep can produce more metabolites related to the organismal energy supply and immune regulation, and these differential metabolites are associated with purine metabolism, amino sugar, and nucleotide sugar metabolism, Fatty acid biosynthesis, and mineral absorption pathways, which are involved in the regulation of rumen growth and development and gastrointestinal homeostasis. Correlation analysis revealed that Tibetan sheep rumen microbes and their metabolites synergistically regulated VFAs and epithelial morphology, enhanced their energy metabolism, and reduced oxidative stress.

## Data Availability

The data sets presented in this study can be found in the NCBI Sequence Read Archive (SRA) under accession number PRJNA1135557.

## Footnotes

^1^www.biocloud.net
