# Supplementary material for: Unique rumen micromorphology and microbiota–metabolite interactions: features and strategies for Tibetan sheep adaptation to the plateau
Source: Front Microbiol. 2024 Oct 9;15:1471732. doi: 10.3389/fmicb.2024.1471732 (PMC11496609; doi:10.3389/fmicb.2024.1471732)
Supplement: Supplementary file 1 [file Data_Sheet_1.docx]

**Table S1**

Nutrient composition of forage in Gannan

| Indicators/Periods | *Poa poophagorum Bor* | *Poaceae* | *Carex coninux* | *Argentina anserina* | *Geranium platyanthum Duthie* |
| --- | --- | --- | --- | --- | --- |
| Aboveground biomass (g/m^2^) | 455.30 | 565.00 | 463.20 | 454.30 | 484.50 |
| Height (cm) | 18.80 | 21.00 | 21.50 | 19.30 | 18.50 |
| Dry matter (%) | 94.53 | 94.70 | 94.48 | 94.64 | 94.69 |
| Crude protein (%) | 11.33 | 11.28 | 10.84 | 11.54 | 11.27 |
| Ether extract (%) | 4.57 | 4.16 | 4.05 | 4.24 | 4.47 |
| Crude Ash (%) | 7.37 | 7.29 | 7.42 | 7.32 | 7.36 |
| Neutral detergent fiber (%) | 58.37 | 59.03 | 58.45 | 57.43 | 57.13 |
| Acid detergent fiber (%) | 34.23 | 34.57 | 33.81 | 33.69 | 33.58 |
| Ca (%) | 0.88 | 0.85 | 0.95 | 0.82 | 0.95 |
| P (%) | 1.17 | 1.69 | 1.30 | 0.96 | 0.91 |
