# Supplementary material for: Unique rumen micromorphology and microbiota–metabolite interactions: features and strategies for Tibetan sheep adaptation to the plateau
Source: Front Microbiol. 2024 Oct 9;15:1471732. doi: 10.3389/fmicb.2024.1471732 (PMC11496609; doi:10.3389/fmicb.2024.1471732)
Supplement: Supplementary file 2 [file Data_Sheet_2.docx]

**Table S2**

Transport gene primers information

| **Gene** | **Primers (5'-3')** | **Length** | **Annealing temperature** | **ID** |
| --- | --- | --- | --- | --- |
| *AE2* | F: AAGATCCCTGAGAACGCCGA  R: AGCAGAAAGAGGAAGCGCAC | 152bp | 60℃ | XM_027969012.1 |
| *DRA* | F: TGTGGCGGCTTCCAGAATTT  R: CACAGGCTTGTTTGGGAGCA | 167bp | 60℃ | NM_001280717.1 |
| *MCT1* | F: GGACTGTGTCATCTGGCAGC  R: TGGGGTCCAACAAGGTCCAT | 134bp | 60℃ | XM_004002335.4 |
| *MCT4* | F: ACGGCTCAGCCTTAGTAAACTTC  R: AATGGAGTTGTGCGAGTTGGT | 144bp | 60℃ | NC_0402252.1 |
| *NHE1* | F: GCTTCTTCGTGGTGTCCCTG  R: CCATGATGCCTGACAGGTGG | 174bp | 60℃ | XM_004005085.4 |
| *NHE2* | F: TTCTTTGTCGTGGGGATCGG  R: CGTGATTGCCATGATGCCTG | 180bp | 60℃ | XM_027967037.1 |
| *β-acting* | F: AGCCTTCCTTCCTGGGCATGGA  R: GGACAGCACCGTGTTGGCGTAGA | 113bp | 60℃ | NM_001009784 |
