# Supplementary material for: Unique rumen micromorphology and microbiota–metabolite interactions: features and strategies for Tibetan sheep adaptation to the plateau
Source: Front Microbiol. 2024 Oct 9;15:1471732. doi: 10.3389/fmicb.2024.1471732 (PMC11496609; doi:10.3389/fmicb.2024.1471732)
Supplement: Supplementary file 3 [file Image_1.pdf]

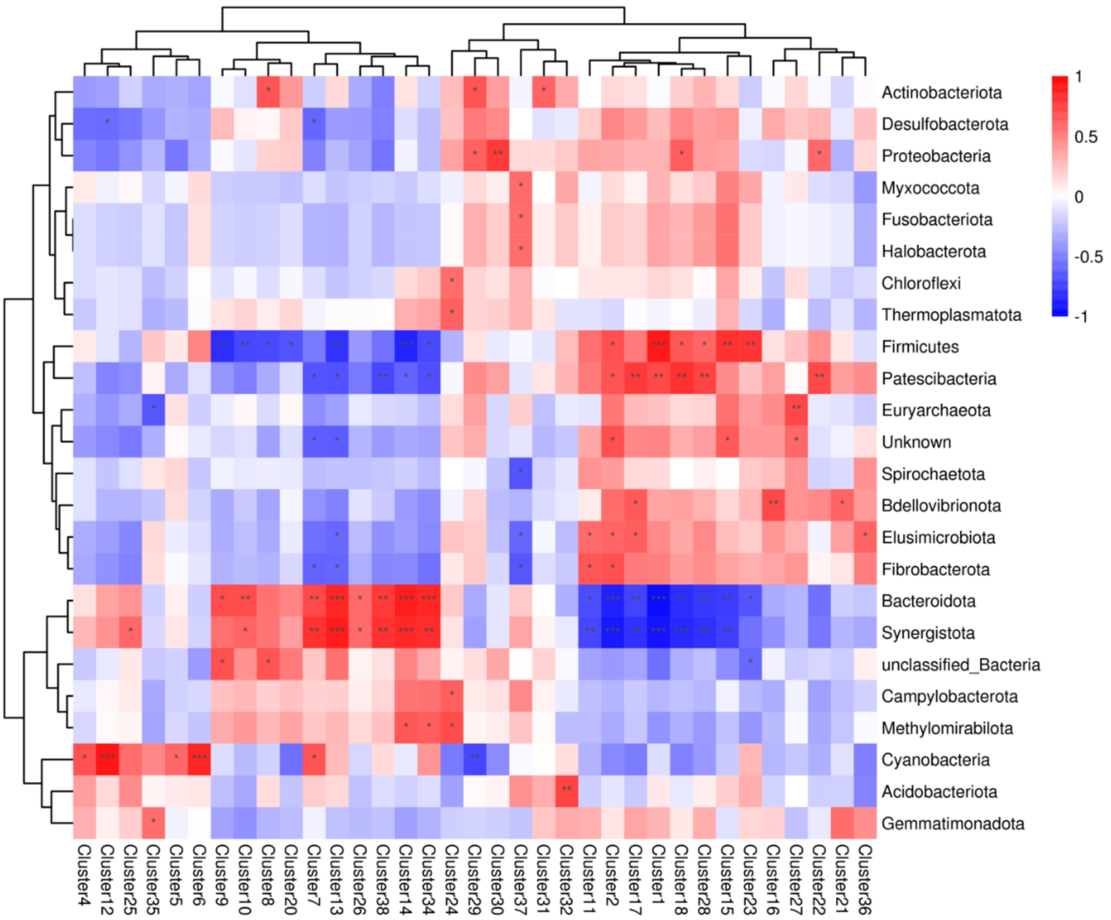

Fig. S1. Heatmap of Metabolite Cluster-Microbial (phylum level) Correlation between Tibetan Sheep and Hu Sheep

Note: *$P < 0.05$, **$P < 0.01$, ***$P < 0.001$.
